# Enhancing response coordination through the assessment of response network structural dynamics

**DOI:** 10.1371/journal.pone.0191130

**Published:** 2018-02-15

**Authors:** Alireza Abbasi, Abolghasem Sadeghi-Niaraki, Mahdi Jalili, Soo-Mi Choi

**Affiliations:** 1 School of Engineering and IT, University of New South Wales (UNSW), Canberra, ACT, Australia; 2 Geoinformation Tech. Center of Excellence, Faculty of Geomatics Eng., K.N. Toosi University of Technology, Tehran, Iran; 3 Dept. of Computer Science and Eng., Sejong University, Seoul, Republic of Korea; 4 School of Engineering, RMIT University, Melbourne, Australia; Bar-Ilan University, ISRAEL

## Abstract

Preparing for intensifying threats of emergencies in unexpected, dangerous, and serious natural or man-made events, and consequent management of the situation, is highly demanding in terms of coordinating the personnel and resources to support human lives and the environment. This necessitates prompt action to manage the uncertainties and risks imposed by such extreme events, which requires collaborative operation among different stakeholders (i.e., the personnel from both the state and local communities). This research aims to find a way to enhance the coordination of multi-organizational response operations. To do so, this manuscript investigates the role of participants in the formed coordination response network and also the emergence and temporal dynamics of the network. By analyzing an inter-personal response coordination operation to an extreme bushfire event, the networks’ and participants’ structural change is evaluated during the evolution of the operation network over four time durations. The results reveal that the coordination response network becomes more decentralized over time due to the high volume of communication required to exchange information. New emerging communication structures often do not fit the developed plans, which stress the need for coordination by feedback in addition to by plan. In addition, we find that the participant’s brokering role in the response operation network identifies a formal and informal coordination role. This is useful for comparison of network structures to examine whether what really happens during response operations complies with the initial policy.

## Introduction

Recent unusual climate change has increased the power and frequency of natural hazards [1]. Responding effectively to such threats to prevent or at least decrease consequent losses is a big challenge for most nations [2]. Such natural or even man-made (e.g., terrorist attacks, airplane crashes) incidents, irrespective of their types, grant uncertainties and risks to the environment and human lives which necessitate instantaneous actions to manage them. Emergency response organizations at national and international levels are responsible for arranging emergency response plans, including standard operating procedures to ensure an appropriate, timely, and effective reaction to such unexpected, dangerous, and serious events. These procedures identify the responsibilities and roles of participants (either individuals or organizations) in the response operation [3].

In large-scale extreme events, such as floods, earthquakes, or bushfires, the participants are often unable to continue with existing planned procedures to deal with the problems because of the uncertain, demanding, and chaotic situation that affects participants’ behavior and performance. Therefore, in these situations, either a single individual or a group of key participants needs to act as coordinator(s) [3]. Coordinating response operations to such massive emergencies is an extremely complex task [4]. Although the involvement of multiple organizations in an Emergency Response Operation (ERO) can enhance the capability to respond properly, coordination of personnel (and resources) from different organizations, especially from different jurisdictions, requires significant effort and skill [4]. Therefore, applying appropriate coordination mechanisms is necessary to facilitate communication among the different parties involved in response operations.

Network analysis has proven to be an appropriate method for studying coordination and emergency disaster management both as a ‘theoretical lens’ and also as an ‘analytical tool’ for a deep understanding of the communication patterns and its dynamics in crises and emergencies [3, 5–9]. It helps to assess the structural dynamics of inter-personal and inter-organizational response networks during an extreme event operation. This can enlighten our understanding of multi-organizational coordination mechanisms, and thus enhance the effectiveness of emergency management procedures.

The main objective of this research is to investigate the dynamics of ERO networks to enhance response coordination, which can lead to improvements in the information flow among the participants involved in the ERO. To do so, an inter-personal response network is mapped by extracting and exploring Kilmore East Fires (one of the devastating fires during the 2009 Victorian Bushfires) focusing on the communication patterns among the key participants in the Incident Management Team. By analyzing the network, we aim to better understand the influential participants’ behaviour and also information flow in the ERO network, which can facilitate the decision making process of emergency managers and policy makers. This is achieved by extracting influential players and information sources. However, we lack accurate and reliable data, tools, and techniques to assess the effectiveness of such response networks during rapidly evolving extreme events [7]. Therefore, addressing the following questions enables us to achieve our goal:

- How can we identify and evaluate the emerging structure of a response network during an extreme event?
- How can we identify certain participants who play coordinating roles in the inter-organizational response network during an extreme event?
- What can be learnt about the participants’ structural dynamics over the emergence of inter-personal response networks during an extreme event?

The remainder of the paper is as follows. Section 2 reviews the relevant literature on coordination research and different mechanisms of coordination, particularly how it has been applied in the context of emergency management and disaster relief. The next section explains the methods used for extracting appropriate types of data (which reflects the interaction among the participants) to form an emergency response coordination network. That is followed by a section assessing the structural dynamics of the response network and also presenting these network structural dynamics over time. Finally, the paper ends with a discussion of the findings and a conclusion.

## Literature review

**Fig 1:**
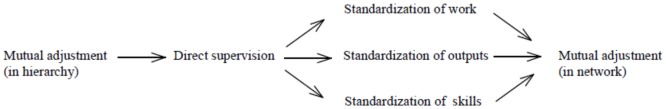
The evolution of coordination mechanisms, from [17].

The study of coordination draws the attention of many scholars across different disciplines including management and organizational science, computer science, economics, and psychology. There is, however, a lack of consensus on the coordination concept, and therefore different definitions have been provided, such as “structuring and facilitation transactions between interdependent components” [10]; “the degree to which there are adequate linkages among organizational parts, i.e., among specific task performances as well as among subunits of the organization, so that organizational objectives can be accomplished” [11]; “the integration or linking together of different parts of an organization to accomplish a collective set of tasks” [12]; “establishing attunement between tasks with the purpose of accomplishing that the execution of separate tasks is timely, in the right order and of the right quantity” [13]; “the additional information processing performed when multiple connected actors pursue goals that a single actor pursuing the same goals would not perform” [14]; and “managing the dependencies between activities” [15]. The last two definitions by Malone et al. focus on ‘why coordination is needed’, compared to the previous definitions emphasising on ‘the desired outcome of coordination’ [16]. Mintzberg’s coordination model [17], depicted in Fig 1, categorizes different coordination mechanisms in organizations and suggests the need for a shift from ‘hierarchy’ to ‘network’ structure.

*“Mutual adjustment”* can be used when few or no formal rules or authority relationships exist. As the environment becomes more complex, organizations coordinate by “*direct supervision”*, with an individual (or a group) in charge to supervise the actions of others. When the supervisor (a person with high authority in the organization) is not capable of making a decision due to the complexity of the environment or rapidly changing conditions, “organisations then have several parallel (and not mutually exclusive) ways of coordinating” [18] by either *standardizing work* (establishing clear procedures for carrying out defined tasks), *outputs* (specifying the result of the work), or *skills* (providing training to the workers). “Standardization typically means control of the agent behavior and minimization of communication” [19]. This supports the argument that different mechanisms should be applied for different scenarios to coordinate resources (including human) for a collective action to accomplish an overall activity.

Dynes and Aguirre [20] introduced ‘*coordination by plan’* and *‘coordination by feedback’* as the main coordination mechanisms in organizations. *Coordination by plan* consists of predetermined programs and activities guiding and regulating the operation of organizations, which have been used massively by traditional emergency organizations such as the army, police, fire, and other emergency agencies. It reflects the processes usually applied to identify a coordinator role in emergency management and the way individuals and organizations should collaborate to control and manage the ERO. In contrast, *coordination by feedback* is about the “learning process and sharing of new information in order to facilitate the mutual adjustments” of parties [21]. When there is a large gap in status and power within an organization, more emphasis is on *coordination by plan*, but in situations when organizational structure becomes so diverse and/or a high level of uncertainty exists in the organizational environment then *coordination by feedback* should be applied [20].

Coordination is one of the fundamental elements directly linked to disaster and emergency management, in particular during the response and relief process [21]. Coordination of the ERO is demanding as it creates intense time pressure and urgency, serious resource deficiencies, significant impact and damage to required infrastructure (e.g., electricity, transportation, and telecommunication networks), and in the worst cases mass casualties [22, 23]. Identifying effective coordination mechanisms to manage interrelated entities (including participants and their assigned tasks and required resources) in extreme events (e.g., bushfires, tsunamis, and earthquakes) is one of the most important challenges for an effective ERO to protect nature and human lives. As shown in Mintzberg’s coordination model, neither a “command and control” model is a suitable coordination mechanism for such complex uncertain dynamic circumstances nor will a single coordination mechanism will work for all cases.

Given the continuous changing status of extreme emergency events, coordination by plan is commonly less ideal and coordination by feedback can be seen as more desirable [21]. In general, in extreme events the rate of communication escalates and changes the organizational structure in a way that requires a more dynamic management structure (coordination by feedback) for effective information exchange [7, 20]. However, it can be argued that better performance in managing emergencies would require using a mix of both types of coordination.

Effective coordination of participants requires a proper communication pattern to share their experience, skills, resources, and equipment for collective collaborative action. The interconnections between participants form a network consisting of participants as nodes (or actors) and links reflecting commination among any pair of nodes. Deep understanding of such inter-personal and inter-organizational networks is necessary to locate information flow and exchange bottlenecks [7]. Facilitating the information flow, through identifying bottlenecks and preventing breakdowns, is crucial in enhancing such a collaborative operation. The flow of resources depends on the structure of the network, i.e. the way different participants are connected to each other. Each form of network structure needs a specific way to control and manage to facilitate flow or avoid breakdowns.

Providing and sharing information is crucial for decision making, particularly in the context of emergency management, as reliable and up-to-date information is necessary for coordinators and other decision makers to maintain and enhance situational awareness. However, the efficient dissemination of information among the involved personnel and organizations in a timely manner is critical for effective coordination. Therefore, investigating the ways the providers and seekers of the information are connected to each other is pivotal for better understanding of their preferred communication structure and patterns. Social network theories often explain the factors affecting the social interaction among members of a community. For instance, social cohesion is claimed to affect reaching an agreement among group members [24] as “members of cohesive subgroups tend to share information and have homogeneity of thought, identity, beliefs, behavior, and even food habits and illnesses” [25].

Although studies such as [8, 9, 21, 26–30] investigate inter-organizational coordination networks during disasters, there are relatively few network analysis studies exploring the emerging network structure of individuals and groups (as sub-networks). Even the existing studies are limited to exploring the change of organizational roles [7, 31], the behaviour of response units from different organizations as coordination clusters [8], and link formation processes among the participants in response networks [3].

## Data and methodology

### Data collection and content analysis

A vast area in the Australian state of Victoria was burnt in February 2009 as a series of bushfires, also called “Black Saturday bushfires”, ignited on Saturday 7 February. These overwhelming and destructive bushfires caused the destruction of over 3,500 structures and 450,000 hectares of land, leaving 414 people injured, 7,562 people displaced, and causing 173 fatalities. One of the largest and devastating series of fires burned a large area around the Kilmore East suburb, 90 kilometers north of Melbourne. The Kilmore East fire was extraordinary and unprecedented with the most severe damage and fatalities (119 people died and 1,242 homes were destroyed).

The data used in this research comes from a content analysis of the reports (transcripts and individual statements) produced by the Victorian Bushfires Royal Commission, which was established shortly after Black Saturday (on 16 February, 2009) to investigate the causes and responses to the bushfires which roared through parts of Victoria in late January and early February 2009. Submissions from Victorian communities were first thoroughly reviewed by the Commission’s legal team, and then considered for the final report that was delivered on 31 July, 2010. All submissions were published on the Commission’s website (www.royalcommission.vic.gov.au) for public use unless agreed otherwise by the Commission. The final report consists of a final summary, proposing 67 “recommendations about changes needed to reduce the risk, and the consequences, of similar disasters in the future”, and four volumes. One of the volumes provides statistics about the 12 main major fires and summarizes the main events of each fire in terms of ‘how it started’, ‘who was involved’, and ‘how emergency services communicated while fighting the fire’. This includes references to the statements of the main personnel involved in the operations, provided to the Commission mainly based on the personal log of events they are required to make during and after operations.

To form an appropriate response coordination network, the data collection involved the following phases:

- Finding the reports related to the special fire (i.e., Kilmore East fires)
- Finding key personnel involved in the response operation
- Extracting the “statement file” for each key Incident Management Team (IMT) participant
- Extracting the interaction among the personnel through mining and reviewing the statement files
- Building, analyzing, and visualizing the response coordination network.

**Fig 2:**
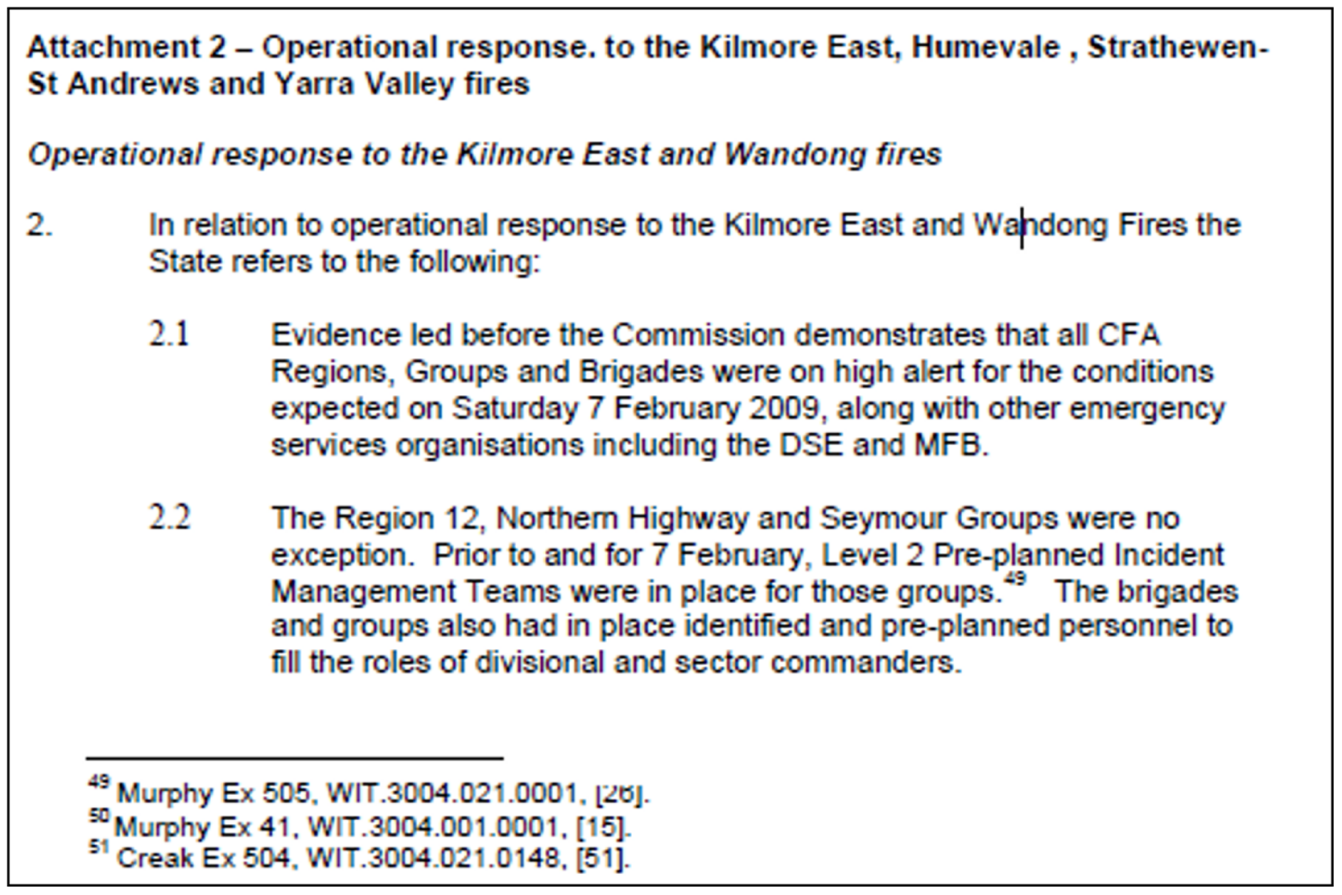
Snapshot of a page of a ‘brief report’ for the “Kilmore East fire” in the 2009 Victorian Bushfires Royal Commission report.

After reading the ‘Kilmore East fires’ section, other relevant documents (such as the ‘brief report’) were identified from the list of references. Reviewing these documents, which summarized the main issues during the incident, enabled us to identify the participants involved in the emergency operation, mainly in the IMT such as the Incident Controller (IC), Deputy Incident Controller (DIC), and Planning Officers (PO), as shown in Fig 2. Then individuals’ statements, listed in the original ‘brief report’ document (see the bottom of Fig 2), were extracted from the repository searching for “Statement of [person name]”.

While mining the individual statements, useful information for each interaction was extracted and stored in a Microsoft Excel file including: who contacted whom (names and roles of the personnel, when available, and the organization they belong to); the time of interaction; their location during communication; content of communication; and the devices and technologies they used. Having the information about involved personnel (nodes) and their interaction (links) enables us to form a network. Finally, UCINET [32], a social network analysis tool, was used for visual representation and measurement of network parameters and participants characteristics (e.g., cohesion; connectedness; structure). As expected, not all the information for all the fields was available but sufficient information (i.e., who communicated with whom and when) were recorded to form a coordination network for further analysis.

### Emergency management system in the Australian state of Victoria

This section presents Victoria’s emergency management planning and operational structure. The Incident Management Team (IMT) is a unit in the Australasian Inter-Service Incident Management System (AIIMS), and is a recognized national system for incident management for fire and emergency service agencies. The IMT comprises a ‘Control’ unit which an Incident Controller (IC) (and for large incidents often a deputy IC) are in charge of to supervise and manage the personnel responsible for other functions such as ‘Planning’, ‘Operations’, ‘Logistics’, ‘Investigation’, ‘Intelligence’, and ‘Public Information’. The IMT forms and operates in close proximity to the incident and in large incidents they may need support from regional units (in which case the IC contacts the Regional Controller).

**Fig 3:**
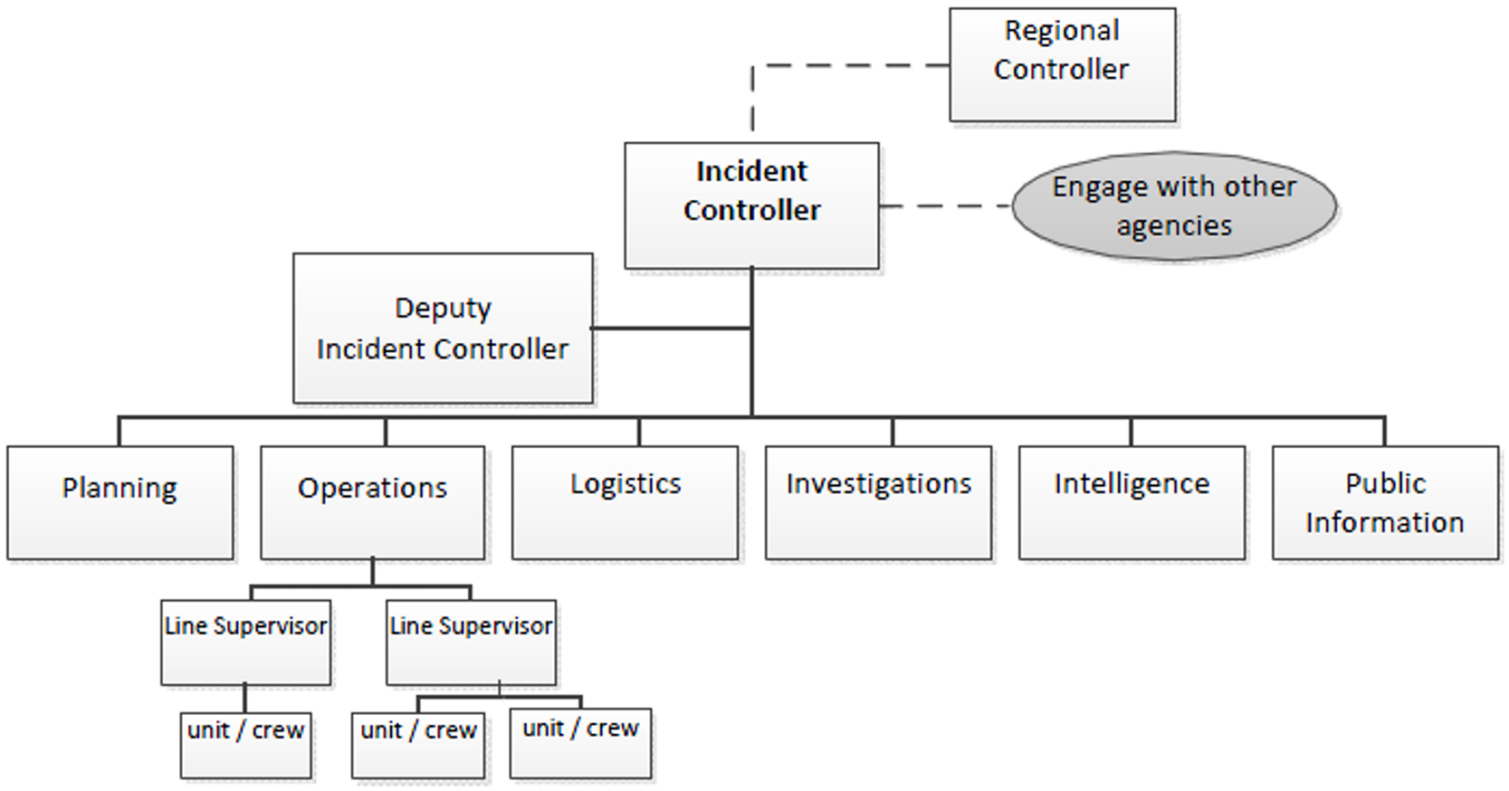
Typical incident management team structure.

As stated in EMMV [33], Victoria uses a three level incident-tier management structure depending on the severity and complexity of the incident. Level 1 incidents “are expected to be resolved through the use of local or initial response resources only; control is limited to the immediate area” and often the IC performs all the functions. Level 2 incidents–which require “a more complex emergency response either in size, resources, or risk”–often need a response generally characterized by “the need for either deployment of resources beyond the initial response; the operations being divided into sectors; the delegation of further IMS functions; or a combination of the above”. Therefore, the IC may retain some managerial functions and other members might be assigned to perform other functions such as operations. Level 3 incidents–more complex situations that might need a more substantial organizational structure to manage the emergency–often involve the formation and cooperation of all incident management functions with separate people responsible for each function [33]. Fig 3 shows an example of a level 3 incident management organizational coordination structure in which the IMT needs to have several personnel running different functions and has to engage with other agencies.

The Kilmore East fire was categorized as a level 3 incident in which different people were responsible for control functions and the IC and deputy IC were coordinating the personnel, resources, and information needed for a proper response among the functions and other agencies, such as a regional control unit which was coordinating parallel fires in different locations at the time.

### Network analysis

A social network is a (social) structure made up of social entities such as people or organizations as nodes and their relationships as ties. Social Network Analysis (SNA) is a mathematical based methodology that views social relationships in terms of network (or graph) theory and examines the structure of relationships between social entities. It is a methodology to analyze networks on different levels (from the whole network structure to dyadic links among pairs) and from different aspects (behavior to attitude). SNA focuses on the structure (pattern) of relations among a set of actors as a core building block of groups and individual behaviors [34]. Although traditional studies in organization science often investigate the effects of individuals’ training on the organization’s performance, SNA perspective focus on individuals’ connections to others inside or outside the organization, which might have an impact on the success of the organization.

During an extreme ERO, a network of people (from diverse organizations) is often formed to share a common goal: to reduce the risk and continuity of the situation for the threatened community [35]. The network’s structure may affect the risk recognition capacity of the participants involved in the response operation process [35]. Connectedness or fragmentation of a network (isolated actors who are disconnected from other actors in the network), may lose influence in the operation of the whole network. The following sub-sections briefly explain the social network metrics used in this study for assessing the structure of emergency response coordination network:

#### Density

Density is a measure which takes into account the aggregate connection among the nodes in a network [36]. Density is the proportion of existing links to all possible links, and is calculated as *n*(*n*-1)/2, where *n* is the number of nodes in the network. Density considers the extent to which all network participants are interconnected and reflects network cohesiveness [37]. During an extreme event, the involved participants (individuals or organizations) are expiating supports (of information and resources) from each other. Thus, the density of the network that comes from the connectivity of the participants in the response coordination network is vital for an effective response to the emergency. Accessibility to information is much easier in highly dense (or fully connected) networks, in which all nodes are linked to others; but such networks are not efficient as they are very costly to establish and maintain.

#### Clustering coefficient

Networks often contain local communities, also called clusters, in which a higher than average number of nodes are densely connected to each other [25]. The simplest way to examine the presence of clusters in a network is to calculate the fraction of “transitive triples”, i.e., ordered triples of nodes A, B, C in which if A-B and B-C are linked then A-C is also linked [38]. Extending this idea, Watts and Strogatz [39] defined clustering coefficient (CC) for node *i* as the density of the sub-network of directly connected nodes to *i*. In other words, assuming node *i* has links to *k*_*i*_ nodes, the clustering coefficient for *i* can be calculated by dividing the number of links that connect the selected *k*_*i*_ nodes to each other by all the possible links among the *k*_*i*_ nodes (i.e., *k*_*i*_ (*k*_*i*_ − 1)/2). Therefore, the CC of a node reflects the likelihood that its directly connected nodes are linked to each other too. The CC of a network can then be simply measured by the average of clustering coefficients of all nodes in the network.

The CC of nodes (i.e., participants) in a coordination network tells us how much participants’ neighbors in the network (directly connected people) communicate with each other. Thus, a low CC value for a network reflects a lower likelihood of communication among the disconnected nodes. This provides opportunity to the participants to intermediate new connections between their direct connections. This might not be a good case for a response operation network as the time available to gain new information and / or resources is very limited and indeed direct links are more efficient.

#### Centrality and network centralization

Assessing the location of nodes in a network is important to understand their role and behavior and also the overall network structure. Node centrality measures (e.g., degree, closeness, and betweenness) assist in identifying the importance of nodes in the network. Although Bavelas [40] originally investigated properties of centrality and suggested several centrality concepts, Freeman [41] formulated the metrics and reported that the centrality of nodes in a network has an impact on their leadership, satisfaction, and efficiency.

Degree centrality is one important measure, and the simplest node centrality measure is measured by the number of adjacent nodes to a node (direct connections). It is noteworthy to mention that a central node is not physically in the center of the network. To examine if a network has a centralized structure, all nodes’ centrality measures have to be taken into account. Thus, network centralization is calculated as a ratio of the sum of the variance of all nodes’ centrality score from the most central node’s score to the maximum possible sum of differences. Therefore, a network’s centralization score specifies how closely the network is organized around its most central nodes [7]. In other words, centralization considers whether network connections and activities are organized around a single node or small group of nodes which refers to the control and power structure of the network [37]. Both density and centralization are concepts referring to different aspects of ‘compactness’ of a network; *density* outlines how cohesive a network is, while *centralization* explains “the extent to which this cohesion is organized around particular focal nodes” [36].

In directed networks, in which the direction of the links between each pair of nodes is important, in-degree (considering the input links: links to a node) and out-degree (considering the output links: links from a node to others) centrality measures for nodes could be used for a more detailed analysis. Consequently, we will use the network in- and out-degree centralizations.

In an emergency coordination network, due to the nature of the command-and-control structure, it is expected that the network structure be centralized around the coordinators of the response operation such as the incident controller. These central coordinators have the power to control the propagation and flow of information in the network.

#### Network hierarchy

Network hierarchy considers the direction of relations among each pair of nodes to assess the proportion of nodes which can reach others but are not reachable from them: i.e. if node A can reach node B but B cannot reach A. This can be regarded as hierarchy in a network—for example, a manager (in an organizational network) may reach his/her subordinate directly or indirectly, but the lower level employee cannot reach his/her manager [42]. Therefore, the degree of network hierarchy is calculated by dividing the number of un-ordered pairs that are symmetrically linked (if A is linked to B, then B is also linked to A) to the maximum possible number of un-ordered pairs where A is linked to B or B is linked A [7].

Network hierarchy exists in networks where the relations among the nodes are built and / or controlled by “status, prestige, or formal authority” [42]. This network metric can be used to investigate the existence of a hierarchy structure in emergency response networks.

## Analysis and results

**Fig 4:**
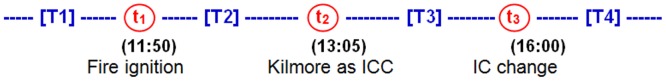
Timeframes for analysis.

In order to investigate the dynamics and emergence of the response network, four periods have been used, as shown in Fig 4. Three significant time points have been identified in regards to the Kilmore East fire: (t_1_) the ignition of the fire at about 11:50 am (on February 7); (t_2_) the time of the establishment of the Incident Control Center (ICC) in Kilmore at 13:05 pm; and (t_3_) the time when the Kilmore East initial Incident Controller (IC) was replaced with a new higher level IC at about 16:00 pm. Therefore, the first period includes all the interactions before t_1_, reflecting the preparedness phase. The second period covers the interactions between t_1_ and t_2_, covering the internal and external communications of the recently formed incident management team. The third period includes the interactions between t_2_ and t_3_, to capture whether the organisational change affected the structure of the coordination response network. The last period covers all the communications which happened after t_3_, mainly covering the interactions until around midnight of February 7 as the available data covering the following days was limited.

### Inter-personal network emergence

During the first day of the Kilmore East fire incident (February 7), 286 interactions among 104 distinct participants (individually involved personnel) were found which indicate the inter-personal communication in response to the incident. Table 1 shows the distinct number of participants (nodes) and the frequency of inter-personal interactions (the number of links), network density, the number of components, and their in- and out-degree network centralization measures for each of the four periods of the Kilmore East fire.

**Table 1:** Cumulative inter-personal coordination network statistics and measures.

	T1	T1-T2	T1-T3	T1-T4
**# of Participants**	**43**	**59**	**78**	**104**
**# of Interactions (Links)**	**73**	**153**	**213**	**286**
**Density (%)**	**4.0**	**4.5**	**3.6**	**2.7**
**Clustering Coefficient (%)**	**34.2**	**46.9**	**60**	**69**
**Hierarchy (%)**	**100**	**97**	**97.5**	**96.8**
**# of Components**	**3**	**1**	**1**	**1**
**The Giant Component Size**	**38**	**59**	**78**	**104**
**Network Centralization (%)**				
**In-Degree**	**5.02**	**3.04**	**2.46**	**3.34**
**Out-Degree**	**10.39**	**7.43**	**5.18**	**4.81**

The results show that the inter-personal response networks in all periods (T1, T1-T2, T1-T3, and T1-T4) have a low density. The second period network has the densest structure but the density of the response network structure decreases over time as a result of new people getting involved without establishing as many connections to others as possible. This reveals that only a few interactions among the participants occurred compared to the maximum possible number of communications. However, apart from during the first period (preparedness phase), the coordination response networks are completely connected, as the number of components is one, meaning there is at least a path between all participants and there are no isolated people in the network. The size of the giant component, which is the number of nodes in the ‘giant component’ (the whole network across the last three periods, increases over time since we used cumulative networks.

It can be seen in Table 1 that the CC is low during the preparedness phase but increases gradually and reaches 69% (almost double that of the first period) during the last period. This reflects that during the preparedness phase there are only a few well-connected clusters (sub-networks of participants) in the network, but as the operation starts more clusters are formed, which probably shows the formation of communication lines among sub-units, such as functions in the IMT. In contrast, the results show a complete hierarchy during the first period which decreases smoothly over time. This shows the existence of a lot of participants who can reach others, but are not reachable by them. This reflects the overall formal authority structure of the response coordination network with ordered relations among the participants in the operation, particularly during the first periods.

**Fig 5:**
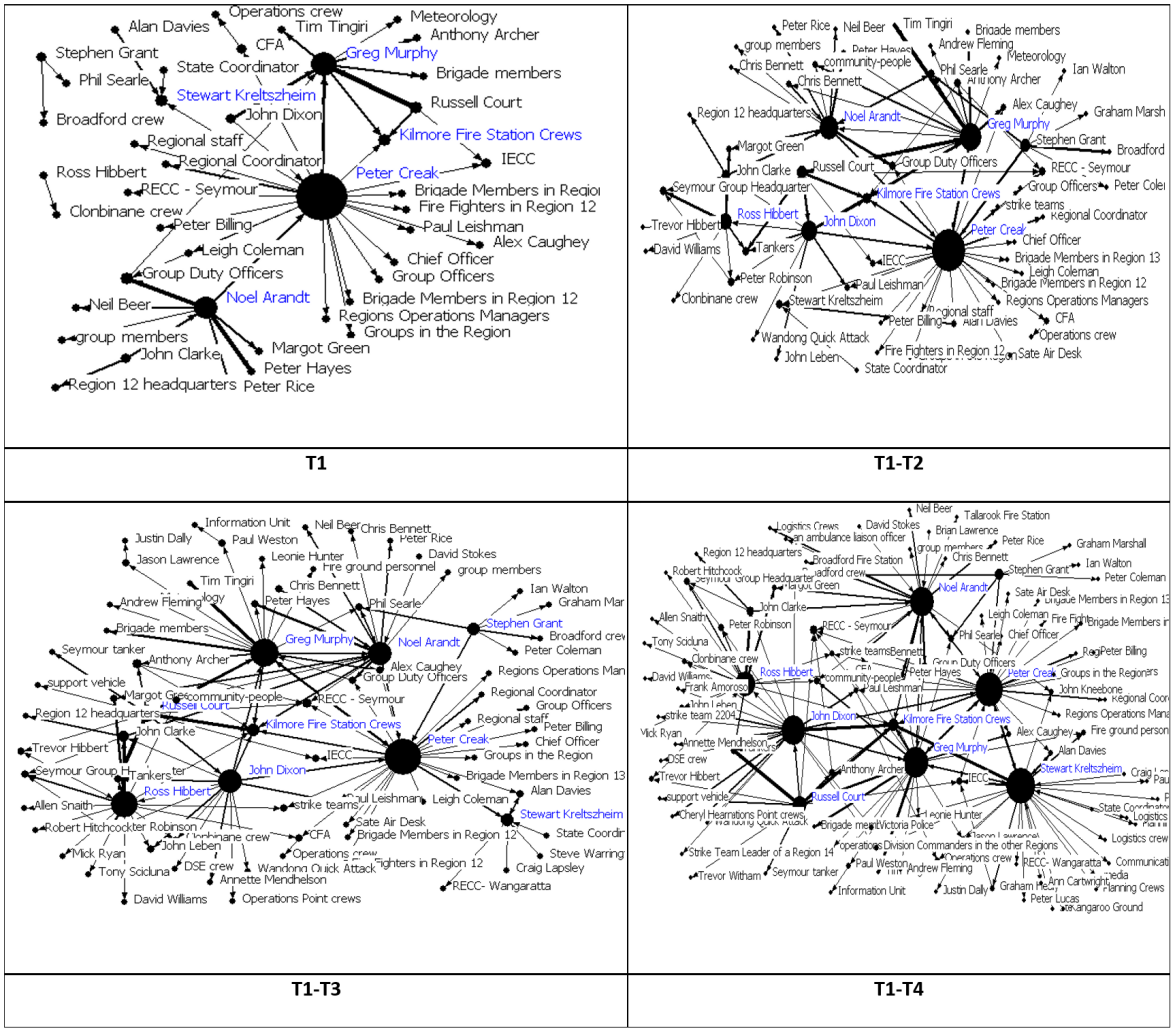
Kilmore East response network (size of the nodes reflects nodes’ degree centrality).

The results for out-degree network centralizations, considering the number of links from each actor to their partners, is depicted in Fig 5 and reveal that although T1 the network structure is relatively centralized, it gets increasingly decentralized during the last periods. This shows that at T1 there is a single participant or a small group of participants in the network who have relatively higher number of out-going links, seeking information or resources. However, over time the number of out-going links for all the participants becomes almost identical as the network structure becomes quite decentralized. This shows that during the preparedness phase (i.e., the first period) few participants seek resources while towards the end of the day (i.e., the last period) the variance of the number of seekers of information decreases (having on average a similar number of requests).

Considering the number of links to participants (in-degree centrality), the overall network structure is quite decentralized, but the response network is more centralized around a few key personnel who provide information (or resources or advice) during the first period, but this fluctuates during the other periods.

Low values for the network centralization measures indicate that the inter-personal response network structure is highly decentralized (considering both in-degree and out-degree centralization). This indicates that there might not be a single coordinator who manages and controls the requests and information flow. However, it is expected that a coordinator should receive a much higher number of requests (i.e., high in-degree) and also provide more information (or advice) (i.e., high out-degree) compared to the other personnel and therefore a high variance between the personnel’s number of communications is expected.

### Participants’ role changes during emergence of response network

To investigate the changes in the positions and/or roles of participants during the emergence of the event, temporal variations of participants’ in- and out-degree centrality measures are assessed. Table 2 shows the top 10 participants who seem to provide information to others as they receive more requests (having high in-degree centrality) from other participants that are seeking resources or any kind of support. Since the degree centrality measure (including in- and out-degree) depends on the number of nodes in a network, a normalized measure (i.e., degree divided by total number of possible links) should be used for comparing nodes and networks with different sizes.

**Table 2:** Top 10 *provider* personnel in the Kilmore East inter-personal coordination network (normalized in-degree centrality).

	T1		T1-T2		T1-T3		T1-T4	
	Participants	In. Deg	Participants	In. Deg	Participants	In. Deg	Participants	In. Deg
**1**	Greg Murphy	0.06	Greg Murphy	0.04	Kilmore Fire St. Crews	0.03	Kilmore Fire St. Crews	0.04
**2**	Group Duty Officers	0.02	Kilmore Fire St. Crews	0.03	Greg Murphy	0.02	Greg Murphy	0.02
**3**	Kilmore Fire St. Crews	0.02	Peter Creak	0.03	Tankers	0.02	Tankers	0.02
**4**	Stewart Kreltszheim	0.02	Tankers	0.02	Peter Creak	0.02	Peter Creak	0.01
**5**	Peter Hayes	0.02	Noel Arandt	0.02	Noel Arandt	0.01	Stewart Kreltszheim	0.01
**6**	Peter Creak	0.01	Tim Tingiri	0.02	Stewart Kreltszheim	0.01	Noel Arandt	0.01
**7**	Noel Arandt	0.01	Phil Searle	0.01	John Clarke	0.01	John Clarke	0.01
**8**	Anthony Archer	0.01	Group Duty Officers	0.01	Margot Green	0.01	IECC	0.01
**9**	Regional Coordinator	0.01	Margot Green	0.01	Tim Tingiri	0.01	Margot Green	0.01
**10**	Tim Tingiri	0.01	Seymour Group Head.	0.01	Group Duty Officers	0.01	Tim Tingiri	0.01

As Table 2 shows, ‘*Greg Murphy*’ (the first IC of the Kilmore East fire station) has the highest in-degree centrality during the first and second periods which means he received more requests from others; this is not the case for other periods. As the network grows over time, ‘*Kilmore Fire St*. *Crews*’ become the highest centralized actors of the network. As shown, participants’ roles (based on their location in the network) do not change as the network evolves.

Table 3 shows the top 10 seeker participants which had more communication with other participants (having high out-degree centrality measures) while seeking support. Although ‘*Peter Creak*’ (Regional Duty Officer) is the most active seeker during the first and second period of the incident, his centrality measure decreases during the following periods. As shown, the top seekers in the first period do not remain in the same position during the following periods, and only a few participants are among the top seekers with a high number of links to others for two or more periods.

**Table 3:** Top 10 *seeker* personnel in Kilmore East inter-personal coordination network (normalized out-degree centrality).

	T1		T1-T2		T1-T3		T1-T4	
	Participants	Out. Deg	Participants	Out. Deg	Participants	Out. Deg	Participants	Out. Deg
**1**	Peter Creak	.110	Peter Creak	.080	Greg Murphy	.055	Stewart Kreltszheim	.051
**2**	Noel Arandt	.081	Greg Murphy	.078	Ross Hibbert	.051	Greg Murphy	.050
**3**	Greg Murphy	.043	Noel Arandt	.060	Peter Creak	.047	John Dixon	.044
**4**	Russell Court	.038	John Dixon	.052	Russell Court	.047	Noel Arandt	.040
**5**	John Dixon	.019	Russell Court	.052	Noel Arandt	.040	Russell Court	.040
**6**	John Clarke	.019	Ross Hibbert	.037	John Dixon	.038	Peter Creak	.039
**7**	Stephen Grant	.010	Stephen Grant	.032	Stephen Grant	.017	Ross Hibbert	.039
**8**	Alan Davies	.010	John Clarke	.014	John Clarke	.008	Stephen Grant	.013
**9**	Group Duty Officers	.005	Anthony Archer	.006	Stewart Kreltszheim	.005	John Clarke	.006
**10**	CFA	.005	Alan Davies	.006	Alan Davies	.005	Alan Davies	.004

Comparing the last column of Tables 2 and 3, which covers the entire response network, we can only see a few participants who are among both providers and seekers. This indicates that there are participants in the network who just send or receive many requests (not both). They are definitely not good candidates to coordinate the response network. Obviously, the participants who have both provider and seeker roles are the ones that are managing the communications by responding or passing the requests from seekers to providers. In order to find these types of participants, we use degree centrality which simply combines the two indicators (out- and in-degree centralities), considering the weight of the links as well. Thus, the highest degree shows the most active actor (that has more communication with other participants either to request or provide information or advice).

Table 4 shows the top 10 active participants in descending order of their sum of (normalized) degree. As shown, ‘*Greg Murphy*’ (the first IC of Kilmore East station) and ‘*Peter Creek*’ (Regional Duty Officer) are among the top 3 active participants (with many interactions) during all four time periods. Looking at Tables 2 and 3, they are also listed among the top providers and seekers, which highlights their role as coordinators with both providing and seeking connections in the response network.

**Table 4:** Top 10 active participants (normalized weighted degree centrality).

	T1		T1-T2		T1-T3		T1-T4	
	Participants	Deg	Participants	Deg	Participants	Deg	Participants	Deg
**1**	Peter Creak	.12	Greg Murphy	.12	Greg Murphy	.08	Greg Murphy	.07
**2**	Greg Murphy	.10	Peter Creak	.11	Ross Hibbert	.07	Stewart Kreltszheim	.07
**3**	Noel Arandt	.09	Noel Arandt	.08	Peter Creak	.06	Peter Creak	.06
**4**	Russell Court	.04	John Dixon	.06	Russell Court	.06	Noel Arandt	.06
**5**	Group Duty Officers	.03	Russell Court	.05	Noel Arandt	.05	John Dixon	.05
**6**	Kilmore Fire St. Crew	.02	Ross Hibbert	.04	John Dixon	.05	Ross Hibbert	.04
**7**	John Dixon	.02	Kilmor Fire St. Crew	.04	Stephen Grant	.03	Russell Court	.04
**8**	John Clarke	.02	Stephen Grant	.03	John Clarke	.02	Kilm. Fire St. Crew	.04
**9**	Stewart Kreltszheim	.02	John Clarke	.02	Stewart Kreltszhe	.02	John Clarke	.02
**10**	Peter Hayes	.02	Tankers	.02	Alan Davies	.02	Tankers	.02

Degree centrality is a local measure which only takes into account the number of direct links to a node. In order to identify the coordinators who manage both the providers and seekers and control the information flow over the network a global measure is required. Betweenness centrality, also proposed by Freeman [41], is a measure which considers the position of a node in the entire network based on the number of times the node lies in the shortest path between any other pair of nodes [43]. Therefore, betweenness centrality is a proxy for the brokerage or intermediating role of a node and it reflects the power and control of the information flow.

Table 5 lists the top five intermediating (or brokering) participants who have the highest betweenness centrality measures during the four time periods. The brokering role clearly expresses the coordinating role of ‘*Peter Creek*’ (the Regional Duty Officer). Interestingly, ‘*Noel Arandt*’, as ‘Deputy Incident Controller’, shows a better brokering role that ‘*Greg Murphy*’, the first Incident Controller of Kilmore East station. Almost the same actors are listed among the top 5 brokering roles over the four periods.

**Table 5:** Top 5 intermediating participants (brokering between actors): Normalized betweenness centrality.

	T1		T1-T2		T1-T3		T1-T4	
	Participants	Bet. %	Participants	Bet. %	Participants	Bet. %	Participants	Bet. %
1	Peter Creak	3.63	Peter Creak	7.90	Peter Creak	4.85	Peter Creak	6.37
2	Noel Arandt	1.80	Noel Arandt	6.70	Noel Arandt	4.76	Noel Arandt	5.41
3	Greg Murphy	1.48	Greg Murphy	4.00	Greg Murphy	3.29	Stewart Krelts	5.03
4	Group Duty Offi	0.29	John Clarke	2.58	John Clarke	1.45	Greg Murphy	4.13
5	CFA	0.23	Ross Hibbert	1.61	Ross Hibbert	1.23	John Clarke	1.25

## Discussion

When an extreme event occurs, the emergency conditions often require individuals from different organizations collaborate collectively in order to respond properly to the event. This requires an active commination channel for sharing and exchanging information and expertise, requesting resources, reporting and/or briefing, and so on [7]. Therefore, a coordination response operation network forms in which individuals from different organizations (nodes) communicate with each other to exchange information, resources, and expertise [21].

While evaluating the emerging structure of the response coordination network, we found that the response network structure varies in each period as a result of the circumstances and consequent requirements. Furthermore, the results reveal that the coordination response network become more decentralized over the evolution of the network. Decentralization tends to offer significant advantages during extreme events [19] by not being bond to the leadership of only one or a few people. Coordination requires a large volume of information seeking and sharing and rapid decision making; however, due to limited human capacity, particularly in the highly tense and stressful circumstances of responding to extreme events such as bushfires, there is unlikely to be an efficient/successful outcome if a single individual is responsible for all coordination, particularly for a long period of time.

While answering the second research question of this study, we have shown that network analysis is a valuable methodology and tool for analyzing the dynamics of communication during response operations to disasters. The measures such as degree and betweenness centrality are shown to be useful to identify the participants’ roles in the operation response network, activeness (in providing and seeking information), and brokering roles respectively. It can be seen that the most central participants (considering both providers and seekers) were just providers or seekers and cannot be considered as good candidates for coordinating the network which requires an intermediating role. Therefore, central participants are not necessarily the most appropriate coordinators. In order to identify the real coordinators in a response network, the sum of out- and in-degree centrality measures is used, which is a surrogate for active participants who have links to many other participants. Such social ties make coordinators more familiar with other actors and builds trust among those involved in these situations. Thus, it is vital for organizations to develop information sharing and exchange policies and procedures for effective and efficient responses to extreme events.

In addition, betweenness centrality, as a proxy for participants who have an intermediating or brokering role, has been used to identify the participants who manage the flow of communication across the whole network. The participants who play the brokering (intermediating) role not only receive requests from some organizations but also respond to them or forward their request to proper actors. They are in a strategic position as they are points of strengths and weaknesses within and among networks. Failure of these nodes leads to the breakdown of the overall network. Therefore, the brokering role, measured by betweenness centrality, identifies formal and informal coordination roles, and is thus useful for comparison of network structures: what is prescribed (in procedures) with what really happens. It has been discussed that these measures can be applied to locate the participants who play a coordinating role during the response operation.

The results of our analysis also present an increase in the rate of communication that creates situations where the communication structure changes rapidly, which is often not accounted for in preparedness plans. This verifies the need for coordination by feedback in addition to by plan [20]. Flexibility in coordination via feedback gives the participants involved in the operation, management, and organizations the capability to respond to unanticipated events appropriately [7]. During an extreme event, flexibility supports organizations and consequently members, helping them be prepared for and to meet the requirements of uncertain conditions [44]. Therefore, flexibility of the coordinating structure in uncertain and dynamic circumstances such as natural disasters should be considered as essential practices for effective response, operation, and recovery efforts [45]. This somewhat answers the last research question; in complex situations such as extreme natural events a single person does not have the capacity to lead communication and the information flow for the whole process, and different participants need to get involved in the leadership and coordinating roles reflecting coordination by feedback. However, more research is required to answer our last research question more precisely.

## Conclusion

This paper investigates the structural dynamics of the response network of Kilmore East fire, the most harmful fire during the February 2009 fires in the Australian state of Victoria. Social network analysis measures are used in order to quantify and distinguish the response networks’ structure, and each participant’s position in the response operation network for each period. Social network analysis can help to understand participants’ roles and response network structural dynamics which are important factors in predicting the evolution of response networks. The method presented and discussed in this research is complementary to previous studies [8, 30, 31] aiming to model coordination dynamics in emergency / disaster response operations.

Using a single dataset can be considered one of the limitations of this study. In order to generalize the results and findings, there is a need to apply these analyses to more data sources in future studies. More samples of data for analysis are needed in order to find the threshold for the optimal network structure metrics (e.g., density, centralization) and the correlation between the network structural changes and network measures and performance. We believe that this approach is not only limited to natural disasters and can be applied to other domains as well.

This study opens an avenue for further analysis of the emerging dynamic structure of inter-personal and inter-organizational response networks during emergencies and disasters in order to facilitate the coordination process and outcome. This research also contributes to emergency and disaster management literature by evaluating dynamic changes of actors and their organizational roles and positions in the inter-personal response networks, which emerges during the extreme events.
